# Outcomes of Fontan patients undergoing combined heart-liver transplantation in pediatric hospitals across the United States

**DOI:** 10.1016/j.jtcvs.2025.08.011

**Published:** 2025-08-14

**Authors:** Grant Chappell, Amir Mehdizadeh-Shrifi, Darren Turner, Alexander Bondoc, Suzanne Evans, Alexander G. Miethke, Gregory Tiao, Meghan M. Chlebowski, Alexander R. Opotowsky, David Lehenbauer, Marco Ricci, Awais Ashfaq, David L. S. Morales

**Affiliations:** a Department of Cardiothoracic Surgery, Cincinnati Children’s Hospital and Medical Center, Cincinnati, Ohio.; b Division of General and Thoracic Surgery, Cincinnati Children’s Hospital and Medical Center, Cincinnati, Ohio.; c Division of Pediatric Gastroenterology, Hepatology & Nutrition, Liver Transplant Program, Cincinnati Children’s Hospital and Medical Center, Cincinnati, Ohio.; d Cardiac Intensive Care Unit, Cincinnati Children’s Hospital and Medical Center, Cincinnati, Ohio.; e Adult Congenital Heart Disease Program, Cincinnati Children’s Hospital and Medical Center, Cincinnati, Ohio.

**Keywords:** combined heart-liver transplant, Fontan-associated liver disease, single ventricle

## Abstract

**Background:**

With increasing numbers of patients surviving Fontan palliation, there is a rise in Fontan-associated liver disease and transplantation strategies inclusive of combined heart-liver transplantation (CHLT). Therefore, we reviewed a combined dataset assessing outcomes of pediatric patients undergoing CHLT.

**Methods:**

Patients undergoing Fontan CHLT between 2010 and 2024 at pediatric hospitals were queried from a merged dataset of the Pediatric Health Information System and United Network for Organ Sharing. Matching (2:1) was completed with Fontan heart-only transplant recipients.

**Results:**

A total of 34 patients underwent Fontan CHLT at 9 pediatric hospitals between 2010 and 2024, 82% (n = 28) after 2019. Almost one-half (47%; n = 16) were age ≤18 years at the time of CHLT, with a median age of 19 years (interquartile range, 15–24 years). More than two-thirds (68%) had hypoplastic left heart syndrome, 38% had double-inlet left ventricle, 26% had double-outlet right ventricle, and 18% had tricuspid atresia. One-third of the patients (n = 11) had a Model for End-Stage Liver Disease/Pediatric End-Stage Liver Disease score of ≤10 at CHLT, with 91% hospital survival, and those with a score>10 had 82% survival. One-year survival was 85% overall, 94% for those age>18 years, and 75% for those age ≤18 years. All deaths occurred before hospital discharge and within 6 months of transplantation, with a mean follow-up of 4 years. Three-year survival was similar in the CHLT and heart transplant -only (HT) groups (85% vs 84%; *P* = .96, log-rank test).

**Conclusions:**

This multi-institutional analysis demonstrates the rapidly increasing numbers of Fontan palliated patients successfully undergoing CHLT in pediatric hospitals. Conditional on surviving to discharge, long-term survival has been excellent, demonstrating a success story in transplantation management for this complex population.

## CHLTCENTRAL MESSAGE

The number of combined heart liver transplants for a failing Fontan performed in pediatric institutions for both adult and pediatric patients is rapidly increasing, with excellent long-term survival.

## Graphical Abstract

Matched Kaplan-Meier survival curves of Fontan combined heart-liver transplantation (CHLT) versus Fontan heart-only transplantation. A, Comparison of CHLT and matched Fontan heart transplant survival. B, Conditional 6-month (6-M) comparison of CHLT to matched Fontan heart transplant survival. Shading represents 95% confidence limit.

With the increasing number of children and young adults successfully surviving Fontan palliation, there has been a relentless rise in Fontan-associated liver disease (FALD),^1,2^ and treatment and transplantation strategies, inclusive of combined heart-liver transplantation (CHLT), continue to evolve.^3^ As pathways are refined, CHLT has become an established treatment in both adult and pediatric hospitals.^4,5^

Initial comparisons of isolated heart transplantation (HT) to CHLT for Fontan patients have been limited, and a multicenter focus on CHLT of children and young adolescents at pediatric hospitals still eludes transplant providers.^6^ Consequently, we sought to investigate the national experience of Fontan CHLT in children and young adults at pediatric hospitals, reporting national trends and outcomes compared to isolated Fontan HT.

## METHODS

This study was deemed exempt from the need for Institutional Review Board approval as a non–human subject study. A waiver of consent was granted (2018–6837; approved October 29, 2018). Patients undergoing Fontan CHLT between 2004 and 2024 at pediatric hospitals were queried from a merged dataset of the Pediatric Health Information System (PHIS) and United Network for Organ Sharing. Patient records were merged via transplantation date and center, age, sex, and admission date, using methods similar to previously published studies.^7^ The PHIS is an administrative database containing clinical and financial details from 49 tertiary care pediatric hospitals in the United States and Canada. Data quality, reliability, and deidentification are ensured through a joint effort between the Child Health Corporation of America and participating hospitals.^8^ Diagnosis and procedures were identified using the International Classification of Diseases, Ninth and Tenth Revisions codes and Clinical Transaction Classification codes. Incomes and costs were adjusted to 2024 using the Consumer Price Index values from the US Census Bureau.^9^ Cost was determined by multiplying the charges by a cost-to-charge ratio for each encounter based on the hospital and discharge year. The cost-to-charge ratio is determined from the official cost reports submitted to Centers for Medicare & Medicaid Services each year. If the cost report is not available, then the hospital is asked to provide the cost-to-charge ratio using a formula provided to them.

**FIGURE E1.**
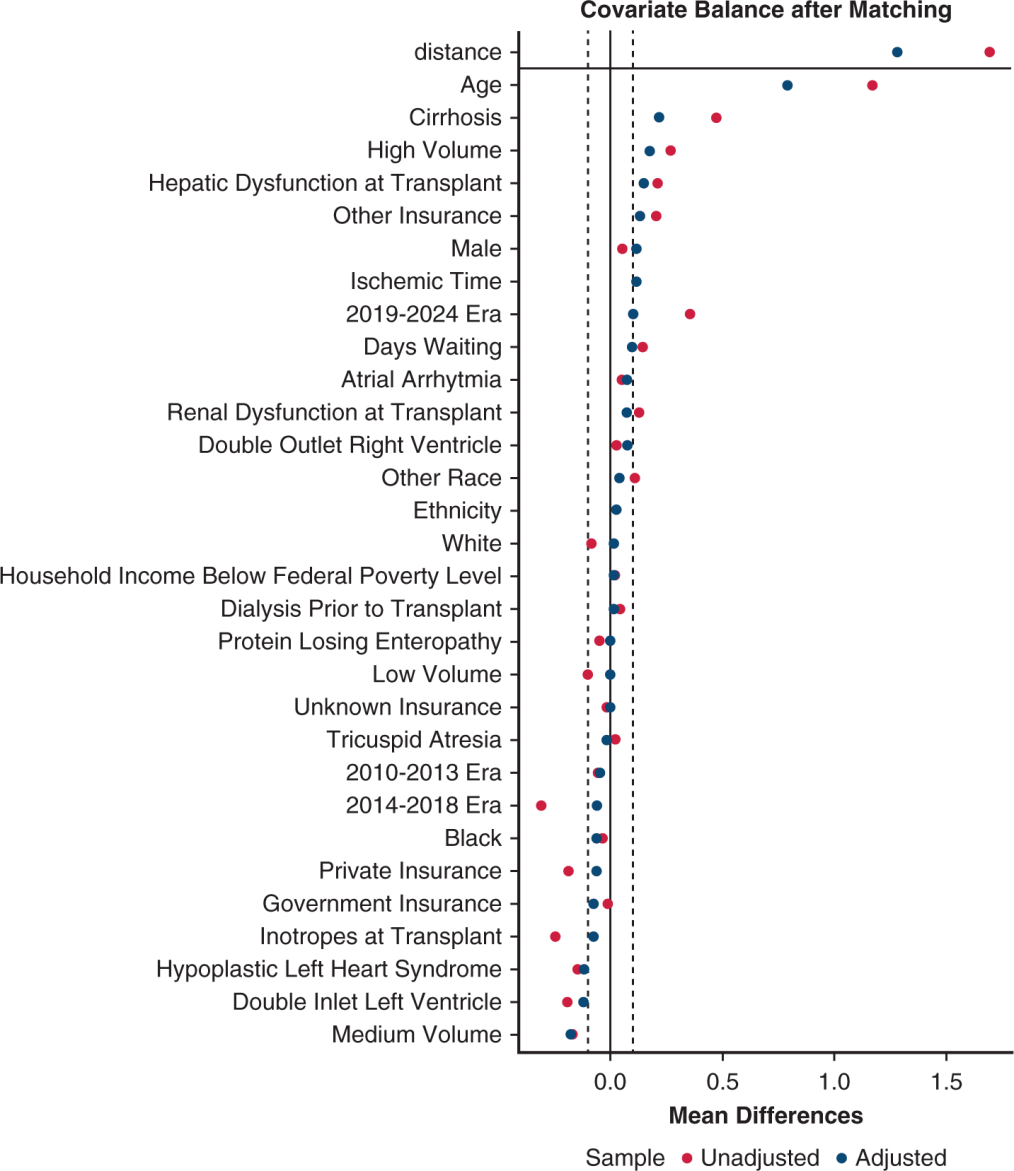
Standardized mean differences of Fontan heart transplant (*HT*) before and after matching with Fontan combined heart-liver transplant (*CHLT*).

Patients were included who underwent CHLT after a diagnosis of hypoplastic left heart syndrome (HLHS), double-outlet right ventricle (DORV), double-inlet left ventricle (DILV), or tricuspid atresia. A subanalysis was performed in patients who were age ≤18 years at the time of transplantation. Participating centers were categorized by total CHLT volume performed for any diagnosis or age from 2004 to 2024. There were 2 low-volume centers (<4 CHLTs performed), 5 medium-volume centers (4–16 CHLTs performed), and 2 high-volume centers (>16 CHLTs performed). A 2:1 matched comparison of Fontan HT and CHLT recipients was conducted using nearest-neighbor propensity score matching (Figure E1, Table E1). Center volume used for matching was stratified into tertiles based on transplant type: for HT patients, HT volume from 2004 to 2024 (<80, low; 80–260, medium; >260, high); for CHLT patients, CHLT volume. We used the previously validated predicted heart mass (PHM) ratio to compare heart size.^10^ For donor PHM/recipient PHM ratio, undersized was defined as a ratio of <0.86; oversized, as a ratio >1.15.

## RESULTS

**FIGURE 1.**
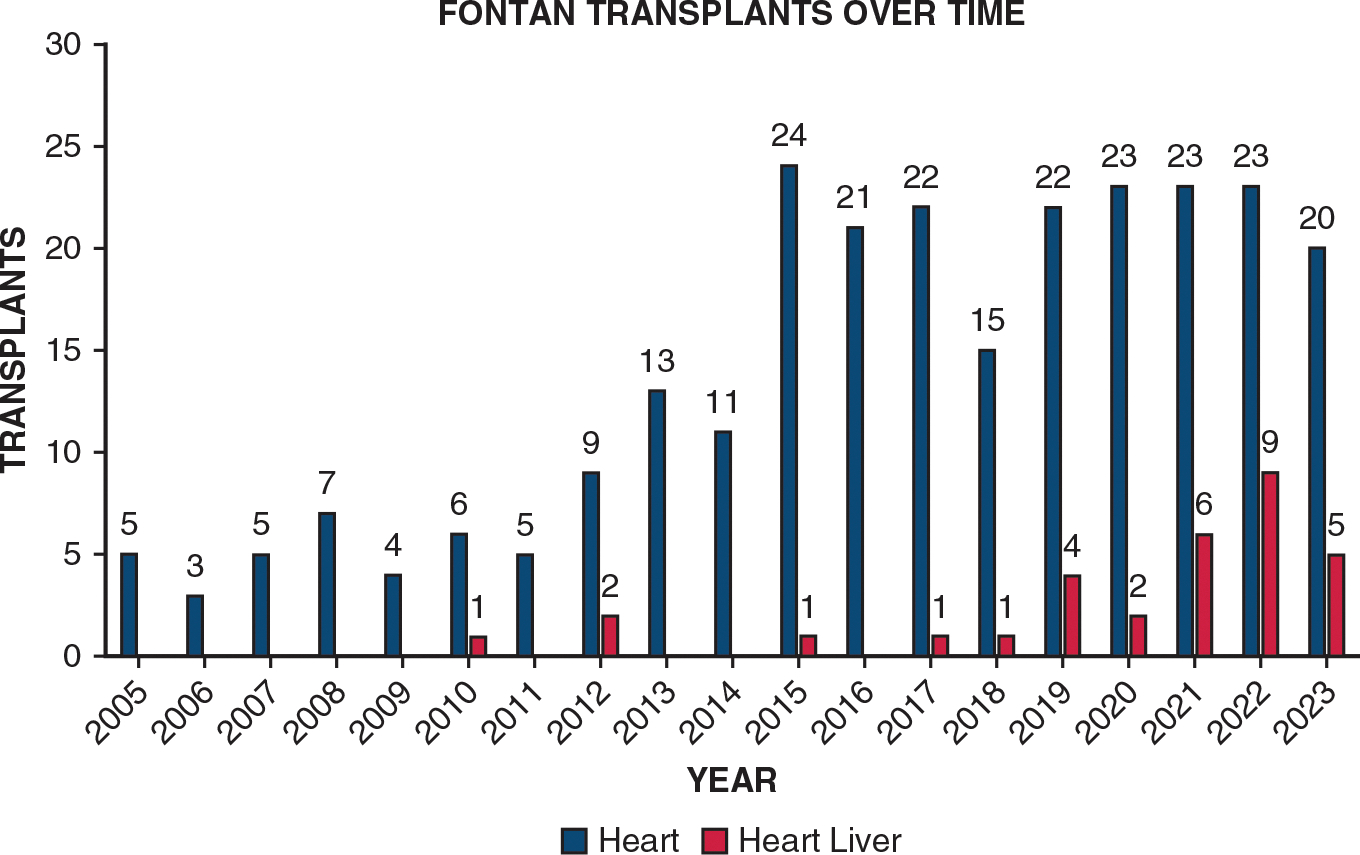
Fontan combined heart-liver transplant and Fontan heart transplant over time.

A total of 34 patients underwent Fontan CHLT at 9 pediatric hospitals. Fontan HT was performed at 31 hospitals; Fontan CHLT, at 9 (29%). Only 3 hospitals performed HTs but did not transplant any Fontan patients. One hospital performed 12 Fontan CHLTs, 2 hospitals performed 6 CHLTs, 1 hospital performed 3 CHLTs, 2 hospitals performed 2 CHLTs, and 3 hospitals performed 1 CHLT. Almost one-half of the patients (47%; n = 16) were age ≤18 years at the time of CHLT. The median age at transplantation was 19 years (interquartile range, 15–24 years; range, 7–46 years). Male patients composed 68% (n = 23) of CHLT recipients, including 75% (n = 12/16) of the pediatric group and 61% (n = 11/18) of the adult group. More than 90% of the patients (n = 31) were from the West (47%; n = 16) or the Midwest (44%; n = 15), with few from the Northeast (6%; n = 2), and South (3%; n = 1). More than four-fifths of the CHLTs (82%; n = 28) were performed between 2019 and 2024, 9% (n = 3) were performed between 2014 and 2018, and 9% (n = 3) were performed between 2010 and 2013, with the first Fontan CHLT in a PHIS pediatric hospital performed in 2010 (Figure 1). More than one-half (53%; n = 18) of the transplantations were at medium-volume CHLT centers, 41% (n = 14) were performed at high-volume centers, and 6% (n = 2) were performed at low-volume centers. Almost one-half (47%; n = 16) had government insurance, including 50% (n = 8) of pediatric patients and 44% (n = 8) of adults, and the median household income was $61,069 (range, $52,608-$93,103). Other than age, demographic data were statistically similar in the pediatric and adult CHLT recipients (Table 1).

Pretransplantation, 59% of the CHLT cohort (n = 20) were inpatients. Over one quarter (26%; n = 9) had atrial arrhytmias. Inotropes were used in 24% (n = 8). The glomerular filtration rate (GFR) was <60 in 18% (n = 6), with 6% (n = 2, both pediatric) requiring dialysis. Three patients (9%; 2 pediatric and 1 adult) were supported by a ventricular assist device (VAD) prior to transplantation. Two of these patients received a dischargeable VAD, one for 39 days and the other for 114 days prior to transplantation. The other patient was placed on percutaneous support for 50 days before removal at time of transplantation. More than two-thirds (68%; n = 23) had HLHS, 38% (n = 13) had DILV, 26% (n = 9) had DORV, and 18% (n = 6) had tricuspid atresia.

Indications for liver transplantation included cirrhosis for 71% of the patients (n = 24), primary liver malignancy for 12% (n = 4), and unknown indications for 18% (n = 6). A diagnosis of cirrhosis was present in 74% (n = 25), 41% (n = 14) had documented ascites, and 12% (n = 4) had protein-losing enteropathy. Less than one-half (47%; n = 16) had a bilirubin concentration >1.2 mg/dL at the time of CHLT. One-third (n = 11) had a Model for End-Stage Liver Disease (MELD)/Pediatric End-Stage Liver Disease (PELD) score ≤10 at CHLT.

Two patients (6%) required extracorporeal membrane oxygenation before CHLT, I of whom continued on ECMO after CHLT. No patient had a tracheostomy before CHLT, although 1 pediatric patient was mechanically ventilated prior to CHLT and required prolonged ventilation (≥96 hours) after CHLT.

Patients were on the waitlist for a median 110 days (range, 48–297 days), including 64 days (range, 46–195 days) in children and 139 days (range, 53–436 days) in adults. Organs were transported a median of 100 nautical miles (range, 16–280 miles) for pediatric CHLT, and 284 miles (range, 125–369 miles) for adult CHLT; and no organ care systems (OCS) for these transplants were identified in our data. Donor characteristics showed a median ischemic time of 3.4 hours (range, 2.8–4.6 hours). Adults received a higher number of undersized hearts (39%; n = 7) compared to pediatric recipients (12%; n = 2), and pediatric recipients received more oversized hearts (56%; n = 9) compared to adults (33%; n = 6), although the difference was not statistically significant (*P* = .209). More than one-half of the pediatric patients (56%; n = 9) underwent concomitant cardiac procedures during CHLT, compared to 44% (n = 8) of adult patients (*P* = .732). Pulmonary artery repair was the most common procedure, performed in 31% (n = 5) of pediatric patients and 33% (n = 6) of adult recipients (*P* = 1) (Table 2).

Outcome analysis showed that less than one-fifth of the patients (18%; n = 6) required dialysis after transplant, while 12% (n = 4) sustained a stroke and 6% (n = 2) were treated for liver rejection (Table 2). ECMO after CHLT was performed in 15% of the patients (n = 5), with a median of 5 days (range, 2–8 days) on ECMO. Under one-half of the pediatric patients (44%; n = 7) and 11% of adults (n = 2) had prolonged ventilation (≥96 hours) after CHLT. Delayed sternal closure was used in 18% (n = 6) of all CHLTs, and 6% of patients (n = 2) required a tracheostomy after CHLT. The median length of stay after CHLT was 62 days (range, 38–100 days). The median cost of hospitalization for CHLT was $868,447 (range, $507,964-$1,159,132). The median total cost of transplantation was $1,484,700 (range, $881,221-$2,240,546) at 1 year after CHLT and $2,139,744 (range, $1,621,376-$2,988,217) at 3 years after CHLT, for a median cost of $331,937 (range, $148,143-$1,113,137) per year of follow-up after transplantation.

**FIGURE 2.**
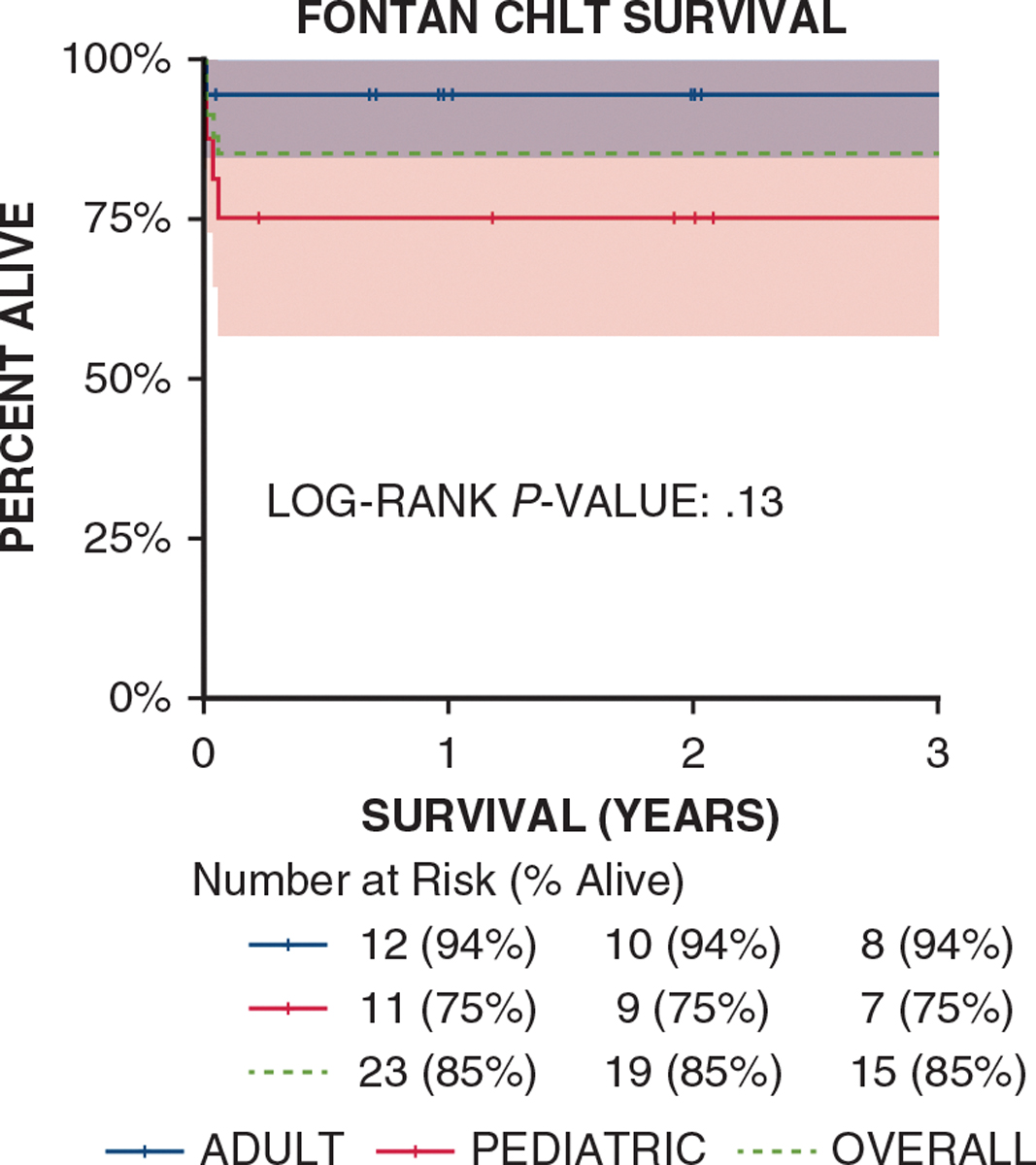
Log-rank *P* value comparing pediatric and adult survival. Shading represents 95% confidence limit. *CHLT*, Combined heart-liver transplant.

Mortality was 15% (n = 5/34) overall, including 25% (n = 4/16) for pediatric patients and 6% (n = 1/18) for adult patients (Kaplan-Meier log-rank *P* = .13 for the difference between pediatric and adult survival) (Figure 2). Three patients died from cardiac complications, 1 patient died from hemorrhage, and 1 patient died from multiple organ failure with liver graft failure. No patient discharged from the hospital after CHLT died, with a mean follow-up of >4 years (range, 0–13 years). At the time of this report, all 3 patients who had a VAD prior to transplant were alive after CHLT, with a mean duration of follow-up of 3 years. Three of the 5 patients on ECMO post-CHLT (60%) survived. Patients with an MELD/PELD score of ≤10 had 91% survival (n = 10/11), including 100% (6 of 6) in adults and 80% (n = 4/5) in pediatric patients, and those with an score>10 had 82% survival (n = 18/22), with 91% survival (n = 10/11) in adults and 73% survival (n = 8/11) in pediatric patients. There was no correlation with mortality and center volume, with no mortality for the 2 patients transplanted at low-volume centers, 17% mortality (n = 3/18) at medium-volume centers, and 14% mortality (n = 2/14) at high-volume centers (*P* = 1).

In the 29 patients who survived longer than 6 months, overall survival was 100% with a median follow-up of 2 years (range, 0.8–4.9 years). One-half (n = 6/12) of the pediatric patients who survived to discharge were readmitted within 90 days after discharge, and two-thirds (n = 8/12) were readmitted within 1 year. Among adults, 35% (n = 6/17) were readmitted within 90 days, and 47% (n = 8/17) were readmitted within 1 year. Infection was the most frequent reason for readmission, accounting for 8 pediatric and 7 adult readmissions within 1 year after transplantation. One pediatric patient and 1 adult patient were readmitted within 1 year for a bile duct obstruction, the only obvious technical complication necessitating readmission. The sole cardiac reoperation performed after CHLT (on postoperative day 10) was in a pediatric patient on ECMO who had chest exploration and was alive almost 4 years after undergoing CHLT. The sole abdominal surgery performed after CHLT was a laparoscopic appendectomy for a pediatric patient at 10 months after CHLT who was alive at last follow-up, 2 years after CHLT.

**FIGURE 3.**
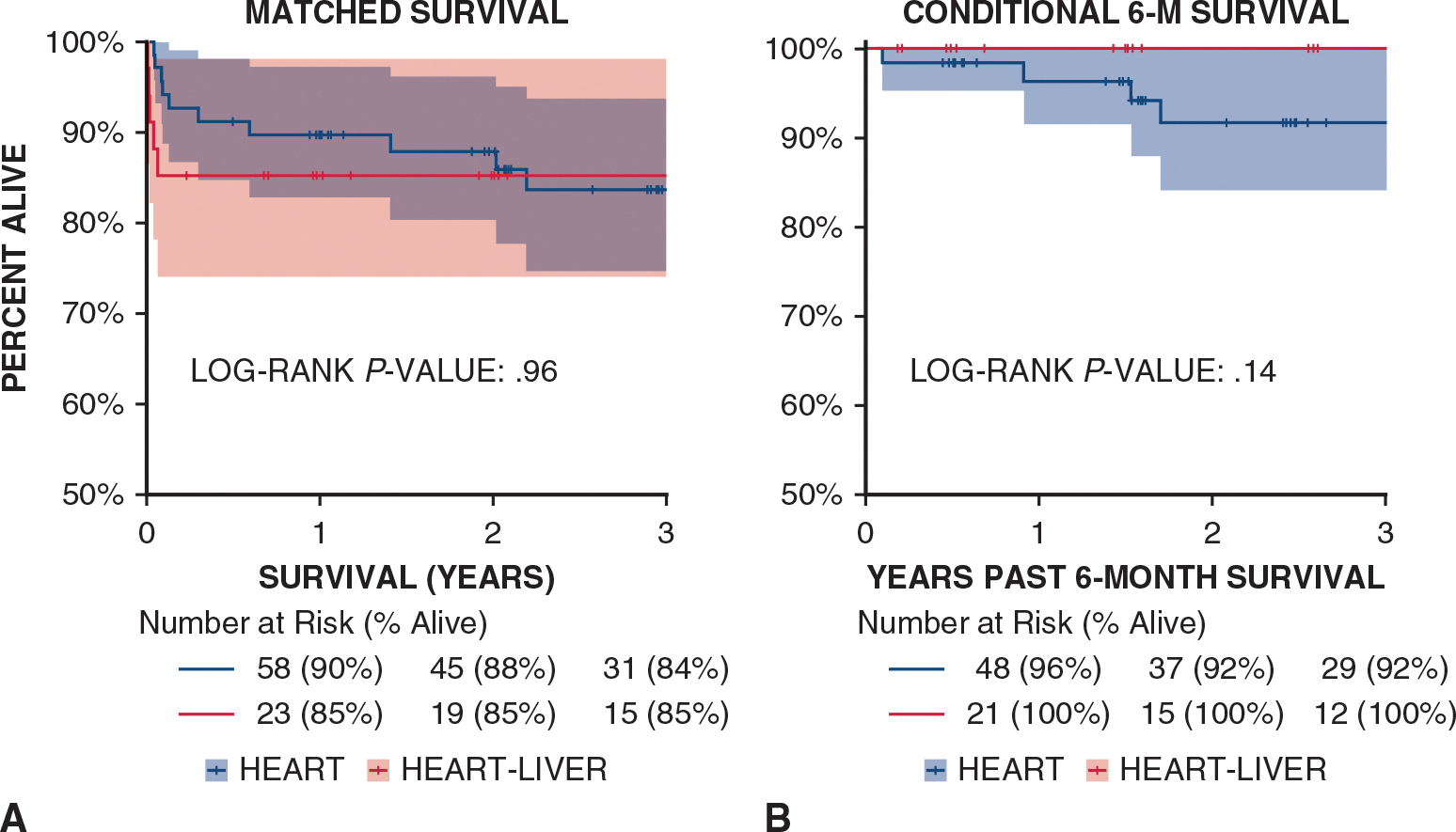
A, Comparison of combined heart liver transplant (*CHLT*) to matched Fontan Heart transplant survival. B, Conditional 6-month (*6-M*) comparison of combined heart liver transplant to matched Fontan Heart transplant survival. Shading represents 95% confidence limit.

A matched comparison of Fontan HT recipients and CHLT recipients revealed the median cost of hospitalization for CHLT was more than double that for HT ($868,447 [range, $507,964-$1,159,132] vs $393,563 [range, $271,615-$997,629]; *P* = .012). However, the cost per year of follow-up post-transplantation was >$100,000 lower for CHLT compared to HT (median, $331,937 [range, $148,143-$1,113,137] vs $468,022 [range, $207,724-$1,018,708]; *P* = .403). The rate of heart rejection at 1 year post-transplantation was 0% in the CHLT group and 19% (n = 13) in the HT group (*P* = .004). Stroke occurred more frequently after CHLT compared to HT (12% [n = 4] vs 3% [n = 2]). Delayed sternal closure also was twice as common after CHLT (18% [n = 6] vs 9% [n = 6]). Three-year survival was similar in the 2 groups at 85% and 84% (Kaplan-Meier log-rank *P* = .96) (Figure 3, A). Among those who survived for >6 months, 3-year survival was 100% for the CHLT group and 92% for the HT group (Kaplan-Meier log-rank *P* = .14) (Figure 3, B). Otherwise, there were no differences between the 2 groups, with similar lengths of stay and rates of dialysis, prolonged ventilation, delayed sternal closure, tracheostomy, and ECMO use and duration (Table 3).

## DISCUSSION

This report describes the national experience of Fontan-palliated children and adults undergoing CHLT at pediatric centers. This multi-institutional analysis revealed a notable increase in the number of CHLT performed in children and adults at these pediatric centers, excellent long-term survival outcomes, comparable results between isolated HT and CHLT, and no post-transplantation mortality after hospital discharge, with a mean follow-up of 4 years. This report aligns with previous comparisons of pediatric and adult hospitals reporting similar outcomes for adults regardless of the treatment center.^11^

Considering significant survival improvements in Fontan-palliated patients, more children are reaching adolescence and adulthood. Therefore, FALD has emerged as a serious concern in this patient population, necessitating transplantation strategies inclusive of CHLT.^2^ Owing to the paucity of data on this topic, we investigated CHLT outcomes in Fontan-palliated children and adults specifically at pediatric hospitals.

The pretransplantation clinical characteristics of our cohort differ from those of previously reported CHLT in Fontan patients, suggesting that centers are identifying patients earlier in the course of heart failure or FALD.^3^ For example, in this study, fewer patients had such Fontan-associated complications as protein-losing enteropathy, cirrhosis, and atrial arrhythmias. This may be related to the infrastructure developed at pediatric centers and is a key driver of the excellent survival trends. This extensive system includes Fontan clinics with scheduled and protocolized follow-up, exercise testing, imaging and catheter studies, as well as an elaborate support system for patients and their families. Moreover, with advanced Fontan-specific clinics and scheduled testing, recognition of functional decline and subsequent referral to advanced therapy (eg, transplantation, VAD) can occur earlier. This type of infrastructure facilitates early identification and management of the failure of the Fontan circulation.^12^ At our center, Fontan management conferences occur weekly and in consultation with hepatologists, supporting frequent, multiorgan surveillance. Additionally, institutional metrics triggering early referrals for advanced therapy are based predominately on current ACTION recommendations and these multidisciplinary conference discussions.^13^

We have noted significant improvement regarding appropriately timed transplant referrals since the opening of the institutional Fontan clinic in 2018. However, even with early identification, referral, and hemodynamic optimization of patients with failing Fontan circulation, advanced operative planning remains essential to provide optimal pathway decision making for children undergoing CHLT in pediatric centers.

Prudence is required when selecting patients for CHLT, because these patients will need 2 valuable organs. Owing to the rarity of Fontan CHLT patients, there are no clear consensus indications for transplantation. At our institution, patients with hepatocellular adenoma or carcinoma, bridging fibrosis diagnosed via biopsy, or the presence of portal hypertension and its sequalae (eg, esophageal varices, splenomegaly, need for multiple paracenteses for ascites) are all indications for including liver transplant for a patient with a failing Fontan. If there is concern that patients are too high risk for transplantation, we advocate for alleviating and optimizing the risk factors known to impact transplantation outcomes, such as nutritional status and renal function using VAD support. For patients who are struggling from a cardiac perspective, resuscitation with a VAD using the ACTION recommendations is essential so that patients are not limping to a major operation such as CHLT with known reversible risk factors.

Waitlist time and distance travelled for organ procurement were almost 3-fold higher in adult CHLT recipients compared to pediatric CHLT recipients at these pediatric centers. We have not used OCS for these transplants as have been used for heart-lung transplantation but have now started listing for CHLT using OCS, and thus an area encompassing the continental United States.^14,15^ However, it should be recognized that the United Network for Organ Sharing (UNOS) OCS data used in the present report were incomplete and could be missing some OCS use.

Notably, one-quarter of pediatric CHLT recipients did not survive to discharge. All the mortality in the cohort was in-hospital, which may reflect the patients’ state going to transplantation or the transplantation itself as major contributing factors. Higher pretransplantation MELD scores correlated with higher mortality rates. Additionally, although not statistically significant, more pediatric patients than adults had pretransplantation risk factors such as VAD use, mechanical ventilation, or dialysis before CHLT. Earlier referrals may be one way to address poor patient status before transplantation. Almost 60% of the present cohort were inpatients going into CHLT. Another strategy would be the use of VADs and aggressive rehabilitation while on a VAD to make patients a better candidate for transplantation.^12,16,17^ We report a small subset of patients on VAD prior to transplantation, which may be underreported given the incomplete mechanical circulatroy support data in UNOS. These strategies could improve hospital survival significantly, which would have a large impact on overall survival given that almost all mortality is early (<6 months). The mid-term survival of this small cohort once discharged from the hospital was 100%, suggesting a protective effect of liver transplantation on heart transplantation.

This multi-institutional report has several limitations, including the retrospective nature of the databases used and selection bias. Furthermore, while this UNOS-PHIS merged dataset increases the amount of information available, these databases are limited in their granularity, clinical reasoning, indication assessment, and delineation of treatment pathways for these highly complex cohorts. Additionally, this study identified a small subset of patients on VAD therapy prior to CHLT. In our single-center experience, we found that VAD use halted, or even corrected, hepatic and renal dysfunction and promoted ventilator weaning in Fontan-palliated patients.^11^ This, along with excellent survival among VAD-bridged Fontan patients and CHLT recipients, has led to increased VAD utilization in the modern era. Although we have reported these findings previously, they are beyond the scope of the present study. Further multicenter investigation with a strong focus on VAD indication is warranted in this population treated at pediatric centers.

In addition, the comparison of Fontan HT patients and CHLT patients was to demonstrate that survival was similar, with the understanding that although grossly matched, these cohorts are different. While not statistically significant, the difference in stroke rate between the 2 groups (12% [n = 4] for CHLT vs 3% [n = 2] for HT) could be related to the fact that patients with worse liver function needing a liver transplant have decreased production of clotting factors, leading to more hemorrhagic strokes after transplantation. This can be a signal that patients undergoing CHLT are more ill than their HT-only counterparts, resulting in more complications because of pretransplantation characteristics.

Nonetheless, there may be a benefit from including the liver when performing transplantation in patients with Fontan failure. None of our CHLT patients had heart rejection, while almost one-fifth of the HT recipients developed rejection. It is well documented that in other multiorgan transplants, liver transplantation provides immunologic protection against rejection,^18^ and this appears to also hold true for CHLT in the present population. Moreover, despite the initial higher cost of transplantation, the post-transplantation yearly average cost is lower for CHLT recipients, which may be a signal that CHLT recipients who survive after admission can do better than their HT counterparts.

**FIGURE 4.**
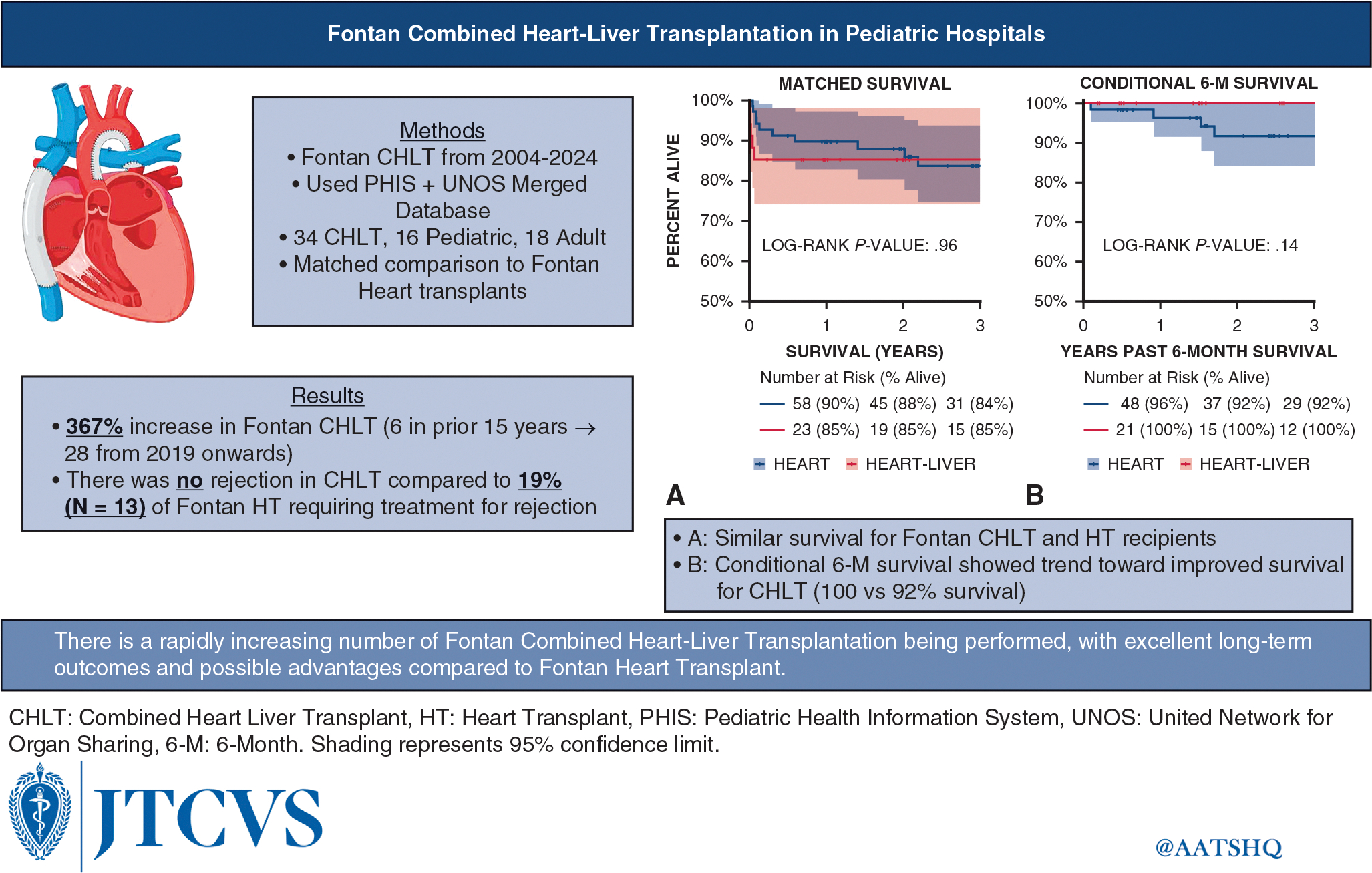
Graphical abstract of the study.

In conclusion, CHLT after Fontan palliation performed at pediatric centers had excellent outcomes for pediatric and adult patients, especially in terms of long-term survival (Figure 4). Considering the significant increase in the use of CHLT and the early concentration of mortality, further refinement and study of referral timing, patient selection, and pretransplantation optimization with VADs is fitting and timely. These excellent post-transplantation survival outcomes are a testament to multidisciplinary collaboration at pediatric centers with established pathways tailored to the transplantation journey of individuals with a Fontan circulation.

## Figures and Tables

**TABLE 1.** Demographics and pretransplantation clinical characteristics of patients undergoing Fontan CHLT

Parameter	All (N = 34)	Pediatric (N = 16)	Adult (N = 18)	*P* value (pediatric vs adult)

Demographics				
Age, y, median [IQR]	19 [15–24]	15 [14–16]	24 [21–31]	<.001
Sex, % (n)				.477
Female	32 (11)	25 (4)	39 (7)	
Male	68 (23)	75 (12)	61 (11)	
Race/ethnicity, % (n)				
Hispanic/Latino	18 (6)	19 (3)	17 (3)	1
White	68 (23)	62 (10)	72 (13)	.717
Black	9 (3)	12 (2)	6 (1)	.591
Other	24 (8)	25 (4)	22 (4)	1
Census region, % (n)				.237
Midwest	44 (15)	31 (5)	56 (10)	
West	47 (16)	62 (10)	33 (6)	
Northeast	6 (2)	6 (1)	6 (1)	
South	3 (1)	0 (0)	6 (1)	
Heart-liver transplant volume, % (n)				.489
Low	6 (2)	12 (2)	0 (0)	
Medium	53 (18)	50 (8)	56 (10)	
High	41 (14)	38 (6)	44 (8)	
Household income, USD, median [IQR]	61,069 [52,608–93,103]	60,031 [51,736–124,192]	62,107 [55,143–74,278]	.727
Household income below the FPL, % (n)	3 (34)	0 (0)	6 (1)	1
Rural patient residence, % (n)	21 (7)	19 (3)	22 (4)	1
Insurance, % (n)				.823
Private	29 (10)	31 (5)	28 (5)	
Government insurance	47 (16)	50 (8)	44 (8)	
Other	24 (8)	19 (3)	28 (5)	
Era, % (n)				.530
2010–2013	9 (3)	13 (2)	6 (1)	
2014–2018	9 (3)	13 (2)	6 (1)	
2019–2024	82 (28)	75 (12)	89 (16)	
Pretransplant clinical characteristics				
Diagnosis prior to Fontan palliation, % (n)				
HLHS	68 (23)	69 (11)	67 (12)	1
DILV	38 (13)	44 (7)	33 (6)	.725
DORV	26 (9)	19 (3)	33 (6)	.448
Tricuspid atresia	18 (6)	19 (3)	17 (3)	1
Indication for liver transplant, % (n)				.6
Cirrhosis	71 (24)	75 (12)	67 (12)	
Primary liver malignancy	12 (4)	6 (1)	17 (3)	
Unknown	18 (6)	19 (3)	17 (3)	
Admitted prior to CHLT, % (n)	59 (20)	56 (9)	61 (11)	1
Waitlist days, median [IQR]	110 [48–297]	64 [46–195]	139 [53–436]	.498
VAD use, % (n)	9 (3)	13 (2)	6 (1)	.600
Inotrope use, % (n)	24 (8)	25 (4)	22 (4)	1
Mechanical ventilation, % (n)	3 (1)	6 (1)	0 (0)	.484
Dialysis, % (n)	6 (2)	13 (2)	0 (0)	.483
Creatinine, mg/dL, median [IQR]	0.9 [0.7–1.0]	0.7 [0.5–0.9]	0.9 [0.8–1.0]	.042
Renal dysfunction (GFR <60 mL/min), % (n)	18 (6)	19 (3)	17 (3)	1
Total bilirubin, mg/dL, median [IQR]	1.2 [0.7–1.8]	1.1 [0.5–1.5]	1.3 [0.8–3.3]	.498
Hepatic dysfunction (bilirubin >1.2 mg/dL)	47 (16)	44 (7)	50 (9)	.745
Albumin, g/dL, median [IQR]	3.9 [3.4–4.1]	3.9 [3.5–4.1]	3.9 [3.4–4.1]	.851
INR, median [IQR]	1.6 [1.2–2.3]	1.51 [1.2–1.8]	1.6 [1.2–2.3]	.585
Atrial arrhythmia, % (n)	26 (9)	25 (4)	28 (5)	1
Cirrhosis, % (n)	74 (25)	75 (12)	72 (13)	1
Protein-losing enteropathy, % (n)	12 (4)	6 (1)	17 (3)	.604
Ascites, % (n)	41 (14)	44 (7)	39 (7)	1
MELD/PELD lab score, median [IQR]	15.0 [9–19]	13.5 [8.8–20]	16 [9–19]	.228
MELD/PELD lab score group, % (n)				1
5–10	33 (11)	31 (5)	35 (6)	
11–40	67 (22)	69 (11)	65 (11)	
Distance organ traveled, mi, median [IQR]	197 [58–341]	100 [16–280]	284 [125–369]	.176
Ischemic time, h, median [IQR]	3.4 [2.8–4.6]	3.4 [2.7–4.5]	3.8 [3.0–4.6]	.603
Heart size donor matching, % (n)				.209
Undersized	26 (9)	12 (2)	39 (7)	
Matched	29 (10)	31 (5)	28 (5)	
Oversized	44 (15)	56 (9)	33 (6)	
Concomitant cardiac procedures (all), % (n)	50 (17)	56 (9)	44 (8)	.732
Pulmonary artery repair	(11)	31 (5)	33 (6)	
Aortic repair	(5)	19 (3)	11 (2)	
Atrial repair	(3)	12 (2)	6 (1)	
Vena cava repair	(2)	6 (1)	6 (1)	

Volume defined as number of transplants performed for the respective organ: low, <4 CHLTs/80 heart transplants (HTs) performed; medium, 4 to 16 CHLTs/80 to 260 HTs performed; high, >16 CHLTs/260 HTs performed. FPL as defined by the US Census Bureau for a family of 4, using income adjusted for inflation to 2024 values using the Consumer Price Index. Laboratory values are those measured at the time of CHLT or the most recent values prior to CHLT. Only 1 patient was age <12 years at CHLT and used the PELD score. Predicted heart mass used for matching, with undersized defined as a <86% donor-recipient ratio and oversized defined as a >115% donor-recipient ratio. *IQR*, Interquartile range; *FPL*, Federal Poverty Level; *HLHS*, hypoplastic left heart syndrome; *DILV*, double-inlet left ventricle; *DORV*, double-outlet right ventricle; *CHLT*, combined heart-liver transplant; *VAD*, ventricular assist device; *GFR*, glomerular filtration rate; *INR*, International Normalized Ratio; *MELD*, Model for End-Stage Liver Disease; *PELD*, Pediatric End-Stage Liver Disease.

**TABLE 2.** Post-transplantation outcomes after Fontan CHLT

Outcome	All (N = 34)	Pediatric (N = 16)	Adult (N = 18)	*P* value (pediatric vs adult)

Length of stay post-CHLT, d, median [IQR]	62 [38–100]	56 [24–123]	65 [49–88]	.498
Liver rejection 1 y after CHLT, % (n)	6 (2)	6 (1)	6 (1)	1
First-time dialysis after CHLT, % (n)	18 (6)	19 (3)	17 (3)	1
Stroke, % (n)	12 (4)	19 (3)	6 (1)	.204
Hospital mortality, % (n)	15 (5)	25 (4)	6 (1)	.164
Follow-up, y, median [IQR]	2 [0.8–4.9]	2 [0.2–3.6]	2 [1.0–4.9]	1
Readmission rate, % (n)	N = 29	N = 12	N = 17	
90 d after CHLT	41 (12)	50 (6)	35 (6)	.471
1 y after CHLT	55 (16)	67 (8)	47 (8)	.452
ECMO				
ECMO pretransplant, % (n)	6 (2)	6 (1)	6 (1)	1
ECMO post-transplant, % (n)	15 (5)	25 (4)	6 (1)	.164
Days on ECMO, median [IQR]	5 [2–8]	7 [5–12]	3 [2–3]	.114
Prolonged ventilation, % (n)	26 (9)	44 (7)	11 (2)	.052
Pericardiotomy/pericardiocentesis, % (n)	3 (1)	6 (1)	0 (0)	.471
Delayed sternal closure, % (n)	18 (6)	13 (2)	22 (4)	.660
Tracheostomy, % (n)	6 (2)	6 (1)	6 (1)	1
Cost (USD), median [IQR]				
Hospitalization cost	868,447 [507,964–1,159,132]	991,138 [667,265–1,221,403]	701,910 [479,782–1,031,233]	.176
Total 1 y after CHLT	1,484,700 [881,221–2,240,546]	1,623,705 [1,168,610–2,572,212]	1,361,179 [687,816–2,020,644]	.176
Total 3 y after CHLT	2,139,744 [1,621,376–2,988,217]	2,452,169 [1,742,676–5,047,283]	2,106,284 [1,453,753–2,669,182]	.447
Cost per year of follow-up	331,937 [148,143–1,113,137]	474,623 [165,317–13,207,728]	258,384 [156,556–874,763]	.176

Cost adjusted for inflation to 2024 values using the Consumer Price Index. Prolonged ventilation defined as invasive ventilation ≥96 hours after surgery. *CHLT*, Combined heart-liver transplant; *IQR*, interquartile range; *ECMO*, extracorporeal membrane oxygenation.

**TABLE 3.** Matched CHLT versus HT in Fontan patients

Matched outcome	CHLT group (N = 34)	HT group (N = 68)	*P* value

Length of stay post-transplant, d, median [IQR]	62 [38–100]	51 [23–108]	.095
First-time dialysis after transplant, % (n)	18 (6)	16 (11)	.776
Heart rejection requiring treatment 1 y after transplant, % (n)	0 (0)	19 (13)	.004
Stroke, % (n)	12 (4)	3 (2)	.075
Mortality, % (n)	15 (5)	16 (11)	1
Follow-up, y, median [IQR]	2 [0.8–4.9]	2.9 [1.1–5]	.403
Readmission rate, % (n)	N = 29	N = 62	
90 d after transplant	41 (12)	39 (22)	.987
1 y after transplant	55 (16)	53 (16)	1
ECMO			
ECMO pretransplant, % (n)	6 (2)	13 (9)	.328
ECMO post-transplant, % (n)	15 (5)	16 (11)	1
Days on ECMO, median [IQR]	5 [2–8]	6 [4–9]	.878
Prolonged ventilation, % (n)	26 (9)	21 (14)	.616
Pericardiotomy/pericardiocentesis, % (n)	3 (1)	4 (3)	1
Delayed sternal closure, % (n)	18 (6)	9 (6)	.208
Tracheostomy, % (n)	6 (2)	1 (1)	.257
Cost, USD, median [IQR]			
Hospitalization cost	868,447 [507,964–1,159,132]	393,563 [271,615–997,629]	.012
Total 1 y after CHLT	1,484,700 [881,221–2,240,546]	1,312,499 [634,894–2,238,275]	.403
Total 3 y after CHLT	2,139,744 [1,621,376–2,988,217]	1,811,480 [1,081,863–2,950,078]	.353
Cost per year of follow-up	331,937 [148,143–1,113,137]	468,022 [207,724–1,018,708]	.403

Cost adjusted for inflation to 2024 value using the Consumer Price Index. Prolonged ventilation defined as invasive ventilation ≥96 hours after surgery. *CHLT*, Combined heart-liver transplant; *HT*, heart transplant; *IQR*, interquartile range; *ECMO*, extracorporeal membrane oxygenation.

**TABLE E1.** Pretransplantation characteristics of unmatched and matched Fontan transplants

Variable	CHLT (N = 34)	Unmatched heart (N = 237)	Unmatched SMD	Matched heart (N = 68)	Matched SMD

Male sex, % (n)	68 (23)	62 (148)	0.109	59 (40)	0.184
Race/ethnicity, % (n)					
White	68 (23)	76 (180)	0.185	66 (45)	0.031
Black	9 (3)	12 (29)	0.111	13 (9)	0.141
Other race	24 (8)	12 (29)	0.298	21 (14)	0.071
Hispanic/Latino	18 (6)	15 (35)	0.078	16 (11)	0.039
Standardized age (SD)	0.54 (1.03)	−0.18 (0.82)	1.372	−0.27 (0.87)	0.855
Diagnosis, % (n)					
HLHS	68 (23)	82 (194)	0.332	79 (54)	0.269
DORV	26 (9)	24 (56)	0.066	16 (11)	0.253
DILV	38 (13)	57 (136)	0.391	52 (35)	0.269
Tricuspid atresia	18 (6)	15 (36)	0.066	18 (12)	<0.001
Households below FPL, % (n)	9 (3)	7 (16)	0.077	7 (5)	0.054
Insurance type, % (n)			0.672		0.367
Government insurance	47 (16)	48 (114)		51 (35)	
Other insurance	24 (8)	3 (8)		10 (7)	
Private insurance	29 (10)	48 (113)		38 (26)	
Unknown insurance	0 (0)	1 (2)		0 (0)	
Era, % (n)			0.843		0.28
2010–2013	9 (3)	14 (33)		15 (10)	
2014–2018	9 (3)	39 (93)		15 (10)	
2019–2024	82 (28)	47 (111)		71 (48)	
Volume, % (n)			0.689		.328
Low volume	0 (0)	10 (24)		0 (0)	
Medium volume	41 (14)	58 (138)		57 (39)	
High volume	59 (20)	32 (75)		43 (29)	
Inotropes prior to transplantation, % (n)	24 (8)	48 (113)	0.521	32 (22)	.198
Standardized days waiting (SD)	−0.07 (1.06)	−0.02 (1.00)	0.147	0.03 (0.98)	.099
Standardized ischemic time (SD)	0.10 (1.07)	−0.02 (0.97)	0.132	−0.05 (0.97)	.154
Dialysis prior to transplant, % (n)	6 (2)	2 (4)	0.221	6 (4)	<.001
Renal dysfunction (GFR <60 mL/min), % (n)	18 (6)	5 (12)	0.405	10 (7)	.213
Hepatic dysfunction (bilirubin >1.2 mg/dL), % (n)	47 (16)	26 (61)	0.454	31 (21)	.336
Cirrhosis, % (n)	71 (24)	23 (55)	1.079	49 (33)	.461
Protein-losing enteropathy, % (n)	12 (4)	17 (39)	0.135	13 (9)	.044
Atrial arrhythmias, % (n)	27 (9)	21 (50)	0.126	21 (14)	.139

Volume defined as number of transplants performed for respective organ: low, <4 CHLTs/80 HTs performed; medium, 4 to 16 CHLTs/80 to 260 HTs performed; high, >16 CHLTs/260 HTs performed. FPL as defined by the US Census Bureau for a family of 4, using income adjusted for inflation to 2024 values using the Consumer Price Index. *CHLT*, Combined heart-liver transplant; *SMD*, standardized mean difference; *HLHS*, hypoplastic left heart syndrome; *DORV*, double-outlet right ventricle; *DILV*, double-inlet left ventricle; *FPL*, Federal Poverty Level; *GFR*, glomerular filtration rate.

## PERSPECTIVE

As the Fontan-palliated population ages, Fontan-associated liver disease has emerged. With treatment strategies and pathways continuing to develop, care must be taken to ensure that these complex patients are receiving care in the appropriate setting. High-volume pediatric hospitals appear to have excellent outcomes for both pediatric and adult patients in this high-risk group.

## Abbreviations and Acronyms

CHLT: combined heart-liver transplantation
DILV: double-inlet left ventricle
DORV: double-outlet right ventricle
ECMO: extracorporeal membrane oxygenation
FALD: Fontan-associated liver disease
GFR: glomerular filtration rate
HLHS: hypoplastic left heart syndrome
HT: heart transplantation
MELD: model for end-stage liver disease
OCS: organ care system
PELD: pediatric end-stage liver disease
